# Understanding drug-related mortality in released prisoners: a review of national coronial records

**DOI:** 10.1186/1471-2458-12-270

**Published:** 2012-04-04

**Authors:** Jessica Y Andrews, Stuart A Kinner

**Affiliations:** 1 Centre for Population Health, Burnet Institute, 85 Commercial Road, Melbourne, VIC, Australia; 2 School of Population Health, University of Queensland, Herston Road, Herston, QLD, Australia; 3 School of Public Health and Preventive Medicine, Monash University, 85 Commercial Road, Melbourne, VIC, Australia

## Abstract

**Background:**

The prisoner population is characterised by a high burden of disease and social disadvantage, and ex-prisoners are at increased risk of death following release. Much of the excess mortality can be attributed to an increased risk of unnatural death, particularly from drug overdose; however, relatively few studies have investigated the circumstances surrounding drug-related deaths among released prisoners. This study aimed to explore and compare the circumstances of death for those who died from accidental drug-related causes to those who died from all other reportable causes.

**Methods:**

A nationwide search of the Australian National Coroners Information System (NCIS) was conducted to identify reportable deaths among ex-prisoners from 2000 to 2007. Using a structured coding form, NCIS records for these cases were interrogated to explore causes and circumstances of death.

**Results:**

Coronial records for 388 deceased ex-prisoners were identified. Almost half of these deaths were a result of accidental drug-related causes (45%). The majority of accidental drug-related deaths occurred in a home environment, and poly-substance use at or around the time of death was common, recorded in 72% of drug-related deaths. Ex-prisoners who died of accidental drug-related causes were on average younger and less likely to be Indigenous, born in Australia, married, or living alone at or around the time of death, compared with those who died from all other reportable causes. Evidence of mental illness or self-harm was less common among accidental drug-related deaths, whereas evidence of previous drug overdose, injecting drug use, history of heroin use and history of drug withdrawal in the previous six months were more common.

**Conclusions:**

Drug-related deaths are common among ex-prisoners and often occur in a home (vs. public) setting. They are often associated with use of multiple substances at or around the time of death, risky drug-use patterns, and even among this markedly disadvantaged group, extreme social disadvantage. These findings reflect the complex challenges facing prisoners upon release from custody and indicate a need to consider drug overdose within the wider framework of ex-prisoner experiences, so that preventive programmes can be appropriately structured and targeted.

## Background

Studies in Australia and elsewhere have identified a markedly increased risk of death in ex-prisoners, compared with the general population, particularly in the first month following release [1-6]. This excess mortality is predominantly due to an increased risk of unnatural death, often due to drug-related causes [1-8]. While many studies have documented excess risk of drug-related mortality after release from custody, few have investigated the circumstances surrounding drug-related deaths among released prisoners.

It is often assumed that excess drug-related mortality is primarily due to reduced drug tolerance after a period of relative abstinence during imprisonment [9,10]. Consistent with this, there is evidence that undergoing drug detoxification within the previous year is positively associated with overdose, with the inverse true of methadone maintenance [11,12]. However, imprisonment does not necessarily imply abstinence from drug use [13]. Concurrent use of multiple drugs has also been identified as a key risk factor for drug-related deaths more generally [6,14-16], although few studies have been able to assess the role of poly-drug use in deaths among ex-prisoners. For released prisoners, the risk of death may be further compounded by chronic disease, mental illness and socio-economic disadvantage -- characteristics that are common in this population [17-19].

In the Australian context, studies examining factors associated with risk of death among ex-prisoners have typically utilised data linkage methods, matching correctional records with a jurisdictional or national death register [20]. However, this approach is limited by the availability of only basic demographic and criminogenic information, rendering it unsuitable for exploring broader risk factors for mortality. In the Australian context, an alternative source of data is the National Coroners Information System (NCIS) which is a centrally administered electronic database of coronial information for all Australian jurisdictions and contains information on deaths occurring from July 2000 onwards [21]. In Australia, a death is generally reported to the coroner where the person died unexpectedly and the cause of death is unknown; the person died in a violent and unnatural manner; the person was “held in care” or custody at the time of death; the person died during or as a result of an anaesthetic; a medical practitioner has been unable to issue a death certificate stating the cause of death; or the decedents’ identity is unknown [21].

A complete NCIS case file includes demographic information, a police narrative of circumstances surrounding death, autopsy and toxicology reports and the coronial finding. The NCIS therefore represents a rich source of data that may allow for a more detailed examination of the circumstances of death post-release. Previous studies exploring drug-related deaths in the community have been able to identify ex-prisoners through coronial records [22,23], although none has focussed specifically on deaths among ex-prisoners. This study used the NCIS to explore the causes and circumstances of drug-related mortality among ex-prisoners nationally. Specifically we aimed to:

1. Determine the underlying cause of death for ex-prisoners identified through a search of the NCIS;

2. Compare the demographic, socioeconomic, health, treatment and drug-use characteristics, and the circumstances of death, for ex-prisoners who died from accidental drug-related causes, to those who died from all other reportable causes; and

3. Examine the toxicological findings for those who experienced an accidental drug-related death.

## Methods

### National Coroners Information System case identification

To identify deaths among ex-prisoners, the NCIS was interrogated using the ‘keyword search’ with the following terms: ‘custodial’, ‘custody’, ‘correctional’, ‘corrections’, ‘gaol’, ‘incarcerated’, ‘incarceration’, ‘inmate’, ‘jail’, ‘probation’, ‘parole’, ‘prison’, ‘prisoner’, ‘remand’ and ‘sentence’. The search was restricted to deaths from 2000-2007, due to the inaccessibility of ICD-10 cause of death codes post-2007. The search is current as of June 2009.

Excluding ‘open’ cases which are those for which the coronial process is ongoing [24], deaths identified through the keyword search were verified for inclusion based on ex-prisoner status. An ex-prisoner was defined as any person in the community who, based on review of NCIS records, was determined to have been held in an adult prison (sentenced or on remand), or to have served home, weekend or periodic detention during their lifetime. Those who had been held in police cells, and cases that referred to a criminal history or previous criminal charges, but did not clearly indicate a previous incarceration episode, were excluded.

### Data collection

NCIS records were coded using a standardised data collection instrument covering demographic characteristics, social circumstances, physical and mental health, substance use, use of treatment services, toxicology and cause of death. Demographic information was extracted from each case file. The circumstances surrounding death were obtained from mostly free-text information contained in accompanying police and toxicology reports, autopsy documents and coronial findings, including where available living circumstances, relationship status, legal employment, general and mental health status, evidence of drug use and route of drug administration, evidence of drug withdrawal, evidence of suicidal intent or self-harm, health treatment status and location of the fatal incident. Although data were abstracted and coded using a standardised template, variation in the contents of coronial records meant that not all variables were coded for all cases.

Underlying cause of death was coded using ICD-10 codes. For the purposes of this study, ICD-10 codes were grouped into accidental drug-related and all other causes based on a scheme proposed by Randall et al [25]. Accidental drug-related death included alcohol-related deaths and all cases where the underlying cause of death was inferred to be directly related to drug use. These included deaths related to mental and behavioural disorders (F10-F16, F19, F55), and accidental causes (X40-X45).

NCIS case information was extracted by two research assistants from 2009–2010. To ensure coding consistency, 1 in 10 records was double-coded throughout the data collection process. Any discrepancies identified in coding were re-coded according to a final consensus. Case ascertainment was not double coded.

### Ethics approval

Ethics approval for the research was obtained from the Victorian Department of Justice Research Ethics Committee, the University of Queensland’s Behavioural and Social Sciences Ethical Review Committee, and the State Coroner Western Australia Coronial Ethics Committee.

### Analysis

First, identified ex-prisoner deaths were characterised in terms of circumstances of death, demographic, socioeconomic, health, treatment, drug-use and toxicological characteristics, using descriptive statistics. Second, characteristics of those who died of accidental drug-related and all other causes were compared using t-tests (for continuous data) and chi-square tests (for categorical data). Where the expected cell count was less than five, Fisher’s exact test was used. Statistical comparisons were performed to the 5% level of significance. All analyses were conducted using SPSS for Windows, Version 17.0 [26].

## Results

### Underlying cause of death

The NCIS search yielded 388 ex-prisoner deaths with an ICD-10 cause of death code available. Of these, 45% (n = 175) were classified as accidental drug-related deaths: 36% (n = 141) were attributed to ‘accidental drug overdose’ and 9% (n = 34) to ‘mental and behavioural disorders due to psychoactive substance use’. The 213 deaths (55%) that were not classified as accidental drug-related were attributed mainly to suicide (30%); injury due to external causes (12%); chronic/infectious disease (9%); poisoning whereby intent was undetermined (2%); and unknown causes (2%).

### Demographic characteristics

Of all detected deaths, the majority was among non-Indigenous males, with a significant proportion homeless and only a small minority employed at the time of death. Compared with those dying from other causes, those dying from accidental drug-related causes were significantly younger, and were less likely to be Indigenous, married, born in Australia, or living alone at or around the time of death (Table 1).

**Table 1:** Demographic and socioeconomic characteristics according to cause of death

**Characteristic (at or around time of death)**	**Accidental drug-related (n = 175) (%)**	**All other causes (n = 213) (%)**	**p-value**
Median age in years	30.0	36.0	<0.001
Male	164 (93.7)	203 (95.3)	0.491
Non-Indigenous	156 (89.1)	152 (71.4)	<0.001
Born in Australia	90 (51.4)	153 (71.8)	<0.001
Homeless	47 (26.9)	42 (19.7)	0.077
Married/de-facto	22 (12.6)	48 (22.5)	0.019
Living alone	28 (16.0)	60 (28.2)	0.006
Employed	29 (16.6)	36 (16.9)	0.903

### Health and treatment characteristics

Overall, the vast majority of cases exhibited poor health profiles and relatively low utilisation of health services at or around the time of death. Compared with other deaths, records of those dying of accidental drug-related causes were significantly less likely to contain reference to a mental health condition or risk of self-harm, but significantly more likely to contain reference to injecting drug use, a history of drug overdose and heroin use, and a record of drug withdrawal in the six months prior to death (Table 2). Among those experiencing an accidental drug-related death, reference to a general health condition (p < 0.001) and to utilisation of alcohol and other drug services (p = 0.004) were more common in those older than 30 years, whereas injecting drug use was more common among those aged 30 years or less (p = 0.006).

**Table 2:** Health, treatment and drug-use characteristics according to cause of death

	**Accidental drug-related (n = 175) (%)**	**All other causes (n = 213) (%)**	**p-value**
General health condition	45 (25.7)	53 (24.9)	0.795
Mental health condition	51 (29.1)	111 (52.1)	<0.001
Recorded risk of self-harm	9 (5.1)	58 (27.2)	<0.001
Health service use (around time of death)	30 (17.1)	42 (19.7)	0.502
General health	16 (9.1)	25 (11.7)	0.601
Mental health	17 (9.7)	19 (8.8)	0.339
Alcohol and other drug	28 (16.0)	22 (10.2)	0.101
Welfare	21 (12.0)	21 (9.8)	0.539
Drug overdose (ever)	17 (9.7)	10 (4.7)	0.031
Injecting drug use (around time of death)	134 (76.6)	31 (14.4)	<0.001
History of heroin use	84 (48.0)	38 (17.7)	<0.001
Opiate substitution treatment (around time of death)	11 (6.3)	10 (4.7)	0.491
Record of drug withdrawal/detox in previous 6 months	27 (15.4)	8 (3.7)	<0.001

### Toxicology reports and circumstances of death

Among accidental drug-related deaths, opioids, particularly heroin and/or morphine, were identified in the majority of cases (82%). Benzodiazepines were identified in approximately half of all accidental drug-related cases, while alcohol, cannabis and methamphetamine were each identified in approximately 20% of these deaths. Tricyclic antidepressants and antipsychotics were identified less frequently (17% and 11% respectively), as was cocaine (7%), which was more likely to be identified in females (p = 0.007). Alcohol was more likely to be identified in those older than 30 years (p = 0.006).

Eighteen percent of all accidental drug-related deaths involved a single drug while 72% involved a combination of alcohol and/or other drugs. In those cases where only one drug was mentioned, heroin/morphine was the most common with 23 cases recorded (74%), followed by benzodiazepines with 4 cases recorded (13%). In those cases where multiple drugs were implicated, nearly all involved opioids (96%), typically in combination with benzodiazepines (63%). Treating all opioids as one drug ‘type’, in 52 cases two drug types were mentioned, and in 74 cases three or more drug types were mentioned. Alcohol was involved in 38 of the 126 cases where a mixture of drugs was involved (30%) (Table 3). There was no significant association between number of drug types involved in death and either gender or age (both p > 0.05).

**Table 3:** Drugs types involved in deaths involving multiple drugs

**Drug combinations identified**	Proportion of deaths (n = 126)* (%)
**2 drug types**	
Opioid(s) + one other drug type	51 (40.0)
Opioid(s) + benzodiazepines	19 (15.1)
Opioid(s) + alcohol	11 (8.7)
Opioid (s) + methamphetamine	6 (3.4)
Opioid(s) + cocaine	5 (4.0)
Opioid(s) + cannabis	4 (3.2)
Opioid(s) + antidepressants	3 (2.4)
Opioid(s) + antipsychotics	3 (2.4)
Two other drug types (excluding opioid(s))	1 (0.8)
**3 or more drug types**	
Opioid(s) + two or more other drug types	70 (56.6)
Opioid(s) + benzodiazepine + other drug type(s)	60 (47.6)
Three or more drug types (excluding opiod(s))	4 (3.2)
**All multiple drug cases**	
Opioid(s) + any other drug type(s)	121 (96.0)
Any two or more drug types (excluding opioid(s))	5 (4.0)

*data missing for 17 accidental drug-related deaths

Information on the location of death was available for 172 accidental drug-related deaths (98%). The most common place for accidental drug-related deaths to occur was in a residential property (67%), followed by a public place (16%), hotel (9%), hospital/care facility (5%) and campground (2%). When compared with all other cases, accidental drug-related deaths occurred more commonly in a residential property and less often in a public place (Table 4).

**Table 4:** Location of death according to cause of death

**Location of Death**	**Accidental drug related death n = 172 (%)**	**All other causes (n = 210) (%)**	**p-value**
Residential property	116 (67.4)	108 (51.4)	0.015
Public place (includes car parks, railway stations and on streets)	27 (15.7)	77 (36.7)	0.043
Boarding house, hotel, backpackers hostel	16 (9.3)	7 (3.3)	0.615
Hospital/residential care facility	9 (5.2)	11 (5.2)	1.00
Caravan/mobile home/campground	4 (2.3)	5 (2.4)	0.992
Military institution/detention centres	0	2 (1.0)	-

## Discussion

Almost half of all ex-prisoner deaths identified through the NCIS were classified as accidental drug-related deaths. Ex-prisoners who died of accidental drug-related causes were on average younger and less likely to be Indigenous, born in Australia, married, or living alone at or around the time of death, compared with those who died from all other reportable causes. Evidence of mental illness or self-harm was less common among accidental drug-related deaths, whereas evidence of previous drug overdose, injecting drug use, history of heroin use and history of drug withdrawal in the previous six months were more common. Consistent with previous research, accidental drug-related deaths in this study were less likely to be associated with psychiatric morbidity or self harm behaviour [16] and more likely to be associated with being single, poly-drug use, history of heroin dependence, overdose and injecting drug use [16,23].

These findings highlight the need to consider drug overdose within the wider context of ex-prisoner experiences so that preventive programmes can be appropriately structured and targeted. For example, in this study the majority of accidental drug-related deaths occurred in a home environment. A previous study in New South Wales also found that the majority of drug-related deaths occurred in a home environment; however, an ambulance was called while the subject was alive in only 10% of cases, and a substantial minority of drug users died alone [22]. One way to reduce drug-related mortality in ex-prisoners may therefore be to train and support peers and family members to recognise, intervene and seek medical assistance in response to an overdose [16,27], possibly in addition to the delivery of take-home naloxone [28-30]. Another effective strategy may be to facilitate access to a safer injecting facility (SIF) for those who return to injecting drug use [31]. This may be achieved by extending the hours of operation for currently operating SIFs, or by funding the operation of more centres. Currently, there is only one SIF operating in Australia, in Kings Cross, Sydney; evaluations have suggested that this SIF has resulted in a reduction in overdose deaths and provided a gateway to drug treatment and counselling [32].

While the majority of accidental drug-related deaths occurred in a home environment, a significant proportion occurred in a public location such as a car park, railway station or the street (16%). A further 9% occurred in temporary accommodation such as boarding houses or hostels. These findings are consistent with a body of research suggesting a link between accidental overdose and use of drugs in unusual settings [33,34]. They may also be understood in terms of the difficulties that many ex-prisoners experience in securing accommodation upon release from custody [35]. These observations provide insight into potential target areas for transitional programmes for prisoners, such as assistance in securing stable and affordable accommodation post-release [15].

In the current study, toxicological findings revealed that the majority of accidental drug-related deaths involved a combination of drugs including opioids, benzodiazepines and alcohol. This is consistent with previous research indicating that a significant proportion of drug-related deaths in ex-prisoners involve concurrent use of multiple psychoactive substances, particularly multiple central nervous system (CNS) depressants [6,27,36]. It is now well recognised that concomitant use of opioids with other CNS depressants, such as benzodiazepines and alcohol, increases the risk of overdose [10]. Indeed, Gossop et al found that for every supplementary drug administered in conjunction with an opioid, the risk of death from opioids nearly doubles [37]. While there has been much focus on reduced tolerance as a driver of drug overdose in ex-prisoners [38,39], these findings indicate that poly-drug use is also important and should be incorporated into multi-faceted interventions. Ex-prisoners may under-estimate the risks posed by decreased tolerance in combination with concomitant use of multiple psychoactive substances. To the extent that this is the case, it may be possible to reduce drug-related deaths in ex-prisoners through educational programs delivered both prior to and after release from custody.

This study had three main limitations. First, data were extracted from largely free-text documents within coronial records and consequently, the information abstracted varied between records, reducing the number of valid observations for particular analyses. Second, it was rarely possible to determine from coronial records when the deceased was released from custody, and thus the amount of time lapsed between release and death. However, recent evidence suggests that ex-prisoner deaths identified through a search of the NCIS are disproportionately drug-related, and occur in the weeks immediately following release [40]. Further, the aim of this paper was to explore the circumstances surrounding drug-related deaths in ex-prisoners, rather than to add to the already substantial body of evidence [1,2,6,27] regarding the time between release and death. As we cannot confirm the amount of time lapsed between release from custody and death, the term ‘ex-prisoner’ in the context of this paper, while literally correct, does not necessarily imply recent release, although it does imply prior imprisonment. Third, our search method would have identified only a small proportion of all ex-prisoner deaths [40], and our conservative decision rule for deeming a death to be of an ex-prisoner may have excluded additional cases from our analysis. Although the sample for this study was therefore a convenience sample, and represented only a small subset of ex-prisoner deaths in Australia, this is the first study to examine drug-related deaths among ex-prisoners nationally, using coronial records.

## Conclusions

Drug-related deaths are common among ex-prisoners and are often associated with poor health and socio-economic adversity. The majority of accidental drug-related deaths involve multiple substances, highlighting the significance of poly-drug use in deaths after release from custody. These findings reflect the complex challenges facing prisoners upon release from custody and indicate a need to consider drug overdose within the wider framework of ex-prisoner experiences. There remains an urgent need for further research to identify modifiable risk factors for drug-related death, and to evaluate transitional programmes to support reintegration and reduce preventable mortality in this vulnerable group.

## Abbreviations

CNS, Central nervous system; NCIS, National coroners information system; SIF, Safer injecting facility.

## Competing interests

The author(s) declare that they have no competing interests.

## Authors’ contributions

JA participated in data collection, performed the statistical analysis and drafted the manuscript. SK conceived the study, and participated in its design and coordination and helped to draft the manuscript. All authors read and approved the final manuscript.
